# Methyl Jasmonate Cytotoxicity and Chemosensitization of T Cell Lymphoma *In Vitro* Is Facilitated by HK 2, HIF-1α, and Hsp70: Implication of Altered Regulation of Cell Survival, pH Homeostasis, Mitochondrial Functions

**DOI:** 10.3389/fphar.2021.628329

**Published:** 2021-02-26

**Authors:** Yugal Goel, Saveg Yadav, Shrish Kumar Pandey, Mithlesh Kumar Temre, Vinay Kumar Singh, Ajay Kumar, Sukh Mahendra Singh

**Affiliations:** ^1^ School of Biotechnology, Institute of Science, Banaras Hindu University, Varanasi, India; ^2^ Centre for Bioinformatics, School of Biotechnology, Institute of Science, Banaras Hindu University, Varanasi, India; ^3^ Department of Zoology, Institute of Science, Banaras Hindu University, Varanasi, India

**Keywords:** methyl jasmonate, tumor cytotoxicity, metabolism, pH homeostasis, chemosensitivitiy

## Abstract

Methyl jasmonate (MJ) displays antineoplastic potential against numerous neoplastic cells. However, several mechanistic aspects of its antineoplastic action against malignancies of T cell origin remain elusive. The present investigation reports the novel targets of MJ and mechanistic pathways of MJ-mediated antineoplastic and chemosensitizing action against tumor cells derived from murine T-cell lymphoma, designated as Dalton’s lymphoma (DL). The present study demonstrates that MJ directly docks to HIF-1α, hexokinase 2, and Hsp70 at prominent binding sites. MJ exhibits tumoricidal action against tumor cells via induction of apoptosis and necrosis through multiple pathways, including declined mitochondrial membrane potential, enhanced expression of ROS, altered pH homeostasis, an elevated level of cytosolic cytochrome *c*, and modulated expression of crucial cell survival and metabolism regulatory molecules. Additionally, this study also reports the chemosensitizing ability of MJ against T cell lymphoma accompanied by a declined expression of MDR1. This study sheds new light by demonstrating the implication of novel molecular mechanisms underlying the antitumor action of MJ against T-cell lymphoma and hence has immense translational significance.

## Introduction

Methyl jasmonate (MJ; IUPAC name Methyl (1R,2R)-3-Oxo-2-(2Z)-2-pentenyl-cyclopentaneacetate), is a methyl ester of jasmonic acid and is ubiquitously present in plants (Cesari et al., 2014). Methyl jasmonate is demonstrated to display a broad spectrum of antineoplastic activities (Cesari et al., 2014; Hong et al., 2016; Zhang et al., 2016; Yousefi et al., 2020). Moreover, MJ is also being used for novel topical treatment for pre-cancerous and cancerous skin lesions (Palmieri et al., 2011)*.* Nevertheless, based on investigations carried out so far, MJ is considered to be and devoid of any significant side effects at therapeutic doses (Cesari et al., 2014; Zhang et al., 2016; Peng and Zhang, 2017; Yousefi et al., 2020). Further, the adjunct therapeutic potential of MJ has also been evaluated in combination with various therapeutic drugs with promising outcomes (Cesari et al., 2014; Yousefi et al., 2020).

The primary mechanism of the antineoplastic activity of MJ is reported to be associated with its ability to dissociate the mitochondrial membrane-bound hexokinase (HK) from voltage-dependent anion channel (VDAC) (Goldin et al., 2008) with cytotoxic consequences in the susceptible malignant cells (Hong et al., 2016; Zhang et al., 2016). Additionally, MJ up-regulates the generation of ROS (Zhang et al., 2016) and shows inhibitory action on the expression of several key metabolic enzymes involved in the oxidative phosphorylation of tumor cells (Li et al., 2017) and pathways of cell death induction (Cesari et al., 2014; Peng and Zhang, 2017; Wang et al., 2018). However, the mechanism(s) implicated in the antineoplastic activity of MJ exhibits tumor-to-tumor variation (Cesari et al., 2014), necessitating investigation of the antineoplastic mechanisms in a tumor-specific manner.

T cell neoplastic disorders are complicated for clinical management (Krok‐Schoen et al., 2018) with a very high occurrence and mortality rate (Bellei et al., 2012; Park et al., 2017). However, little is understood concerning the mechanisms underlying such antineoplastic action of MJ against cells of T cell malignancies. Moreover, to date, HK 2 is the only known main target of MJ. Thus, there is an immediate need to identify the other probable targets of the MJ. Further, the effect of MJ on the expression of HIF-1α, which is considered as the master regulator of tumor metabolism (Nagao et al., 2019), remains unexplored. Nevertheless, HIF-1α is also an upstream regulator of HK 2 (Pezzuto and Carico, 2018). Additionally, the modulatory effect of MJ on Hsp70, which plays a pivotal role as a regulator of tumor cell survival (Tsuchida et al., 2014) and is downstream to HIF-1α (Pezzuto and Carico, 2018), remains unclear. Further, bioinformatics STRING databases strongly indicate the liaison of HIF-1α, HK 2, and Hsp70 in a network of closely linked cooperative molecules involved in regulating tumor metabolism and survival (Szklarczyk et al., 2015). However, a comprehensive investigation of MJ’s effect on the modulation of HIF-1α accompanied by HK 2 and Hsp70 remains to be investigated. Further, the impact of MJ on various critical cellular activities of neoplastic cells, including metabolism, pH homeostasis, chemoresistance, and production of tumor-promoting cytokines, remains mostly unexplored. Hence, these parameters must be examined in detail before clinical applications of MJ against the T cell lymphoma patients. To address these problems, we used a murine transplantable T cell lymphoma designated as Dalton’s lymphoma (DL), which has been extensively used to understand the host-tumor relationship (Chandran et al., 2016; Koiri et al., 2017; Yadav et al., 2018) and mechanisms of the antineoplastic action of various chemotherapeutic drugs (Pandey et al., 2019; Gupta et al., 2020). DL originated in the laboratory of Dr Albert J. Dalton at NCI, Bethesda, United States (Goldie and Felix, 1951; Dunham and Stewart, 1953) as a spontaneous thymoma and was later on adapted for ascitic tumor growth (Klein, 1951).

The availability of crucial protein crystals on protein data banks and their utilization for *in silico* prediction of active sites and molecular docking techniques to supplement the understanding of the molecular mechanisms underlying drug-target interactions (Li et al., 2020) have given new dimensions to the knowledge of the drug-target interactions. Till now, there has been no study in this direction using MJ. Hence, it is essential to generate and investigate the molecular docking data of MJ with critical metabolic and cell survival regulatory targets, which have remained entirely unaddressed so far. Further, *in silico* tools for studying drug-target interactions also provide strong leads for carrying out modulations of drug binding targets (Loging, 2016; Li et al., 2020) for studying the antineoplastic potential of MJ in clinical applications. Indeed, the development of several present-day anticancer regimens on drug repurposing, discovery, and designing is based on the foundations of *in silico* associated inputs (Li et al., 2020).

Given the lacunas cited above, the present study was undertaken to investigate the additional targets of MJ and novel mechanisms underlying its tumoricidal action, focusing on the implication of altered mitochondrial function, pH homeostasis, cell survival and metabolism regulatory molecules, and chemosensitivity. The study also sheds light on the molecular mechanism of MJ docking to crucial metabolic and cell survival regulatory targets.

## Methods

### Reagents and Cells

All reagents used were of tissue culture or analytical grade. Reagents were purchased from Gibco (United States), Himedia (India), Sigma-Aldrich (United States), and Invitrogen (United States). Antibodies against the indicated proteins were obtained from Imgenex (United States), Cell Signaling Technology (United States), Affinity BioReagents (United States), and Sigma-Aldrich (United States). Pathogen-free inbred adult female mice of BALB/c strain were used at 8–12 weeks of age for obtaining thymocytes from the thymi using the standard method. DL cells are maintained by *in vitro* passaging in culture and serial transplantation in mice. Mice use was done as per approval and guidelines of the institutional ethical committee (Approval reference number: BHU/DOZ/IAEC/2018–2019/019 dated January 28, 2019).

### MTT Assay for Estimation of Cellular Metabolism

Metabolic activity was estimated by standard MTT assay as described earlier (Mosmann, 1983) with slight modification. The plates were read on an ELISA plate reader (Labsystems, Helsinki, Finland) at a wavelength of 540 nm.

### Trypan Blue Dye Exclusion Test for Enumeration of Cell Viability

Viable cells were enumerated using the standard trypan blue dye exclusion test as described earlier (Yadav et al., 2017a). Cells that did not exclude trypan blue were considered non-viable. Data are presented as the percent of viable cells.

### Analysis of Apoptotic and Necrotic Mode of Cell Death

Mode of cell death induction was confirmed by enumeration of the percentage of cells showing apoptotic and necrotic features using Wright Giemsa staining and flow cytometry by Annexin-V/PI staining following the method described by Rieger et al., 2011.

### Estimation of Cytotoxicity

Cytotoxicity was assayed as described earlier (Kumar et al., 2013) with slight modifications using MTT assay by using the following formula:

$$\text{\% Cytotoxicity}=\frac{\text{OD of control tumor cells}-\text{OD treated tumor cells}}{\text{OD of control tumor cells}}*100$$

### Cytochrome *c* Release Assay

Cytochrome *c* (Cyt *c*) release was assayed in the mitochondria-free cytosol by Western blotting following the method described by Gewies et al., (2000). The resultant cell lysates were centrifuged (16,000 X g) for 20 min at 4°C, and the supernatant was used for immunoblotting.

### Immunoblot Analysis

The expression level of various proteins in the cell lysates was carried out following the method described by Fido et al., 1995. The images of immunoblots were captured on a gel documentation image analysis system, and the intensity of bands was analyzed by Quantity One software (Bio-Rad, Australia). β-actin was used as an internal control.

### RT-Polymerase Chain Reaction for Expression of mRNA

RT-PCR analysis for mRNA expression of the indicated genes was carried out by one-step RT-PCR cell to cDNA kit (Ambion, United States). The primer sequences for various genes are shown in Supplementary Table S1.

### Enzyme-Linked Immunosorbent Assay for Detection of Cytokines

Standard ELISA was performed to detect the presence of indicated cytokines in culture supernatant following the method described by Da Silva et al., (2002). The absorbance was measured after 10 min at 405 nm by an ELISA plate reader (Lab Systems, Finland).

### Estimation of Intracellular Reactive Oxygen Species

ROS estimation was carried out as described by Furuta et al., (2008). The cells stained with the dye were visualized under fluorescence microscope and Flow cytometer (Nikon, Japan) at a magnification of ×400 and photographed. The staining was quantified by MCID software.

### Measurement of Intracellular pH

Intracellular pH (pHi) was determined following the method of Franck et al., (1996). The emission spectra of the harvested supernatant, after centrifugation, was taken using a spectrofluorimeter (Varian, United States) at an excitation wavelength of 488 nm. Fluorescence intensity was converted into pH value using a calibration curve of the same number of cells incubated in K^+^ buffer of different pH ranges followed by the addition of nigericin (10 µM) and recording of emission spectra at 488 nm.

### *In Silico* Analysis

**Retrieval of MJ 3D structure:** We retrieved 3D structures of MJ from the PubChem database (https://pubchem.ncbi.nlm.nih.gov/). The retrieved structure of MJ (https://pubchem.ncbi.nlm.nih.gov/compound/5281929) was used for docking analysis following conversion to the PDB format and optimization of the same using Discovery studio 3.5 following the method described by Yadav et al., (2017b).

**Retrieval of the 3D structure of target molecules:** The 3D structures of most plausible target molecules, namely: HIF-1α, HK 2, and Hsp70 which have been demonstrated to play a central role in regulating tumor cell metabolism, survival, and progression (Boudesco et al., 2018; Pezzuto and Carico, 2018; Garcia et al., 2019) were retrieved from the protein data bank (https://www.rcsb.org/). HIF-1α (https://www.rcsb.org/structure/5jwp), HK 2 (https://www.rcsb.org/structure/5HEX), and Hsp70 (https://www.rcsb.org/structure/6jpv) of *Homo Sapiens* were extracted based on selection criteria of PDB standard protein BLAST (Berman et al., 2002), displaying the minimum permissible score and query cover in BLAST with Mus Musculus. Details on the retrieved target protein molecules are summarized in the Supplementary Table S2. Details about the retrieved ligands are summarized in Supplementary Table S3. The evaluation of the structural residues was checked using Rampage 2.0 and PDBsum.

**Active binding site prediction and characterization:** Metapocket 2.0 server (https://projects.biotec.tu-dresden.de/metapocket/) was used to predict active sites of the selected 3D structures of the target molecules accompanied by analysis using Discovery studio 3.5.

**Docking analysis:** Docking tool YASARA and Patchdock server with RMSD value 4.0 and default parameter of complex type protein-small ligand mode was used for docking analysis of MJ to its target molecules (HIF-1α, HK 2 and Hsp70). The docked complexes were visualized on Discovery studio 3.5. Strength of docking was analyzed using criteria including calculations of geometric shape complementarity score (GSC-Score), approximate interface area (AI area) through PatchDock along with predictions of dissociation constant (Kd) and binding energy by YASARA (Dulebo et al., 2012). The positive value of energies was taken as an indication of more durable binding.

### Statistical Analysis

Experiments were conducted at least thrice. The Student’s *t*-test analyzed the statistical significance of differences between various test groups. Value of *p* when less than 0.05 was considered significant.

## Results

### Docking Analysis to Predict Targets of Methyl Jasmonate

**FIGURE 1:**
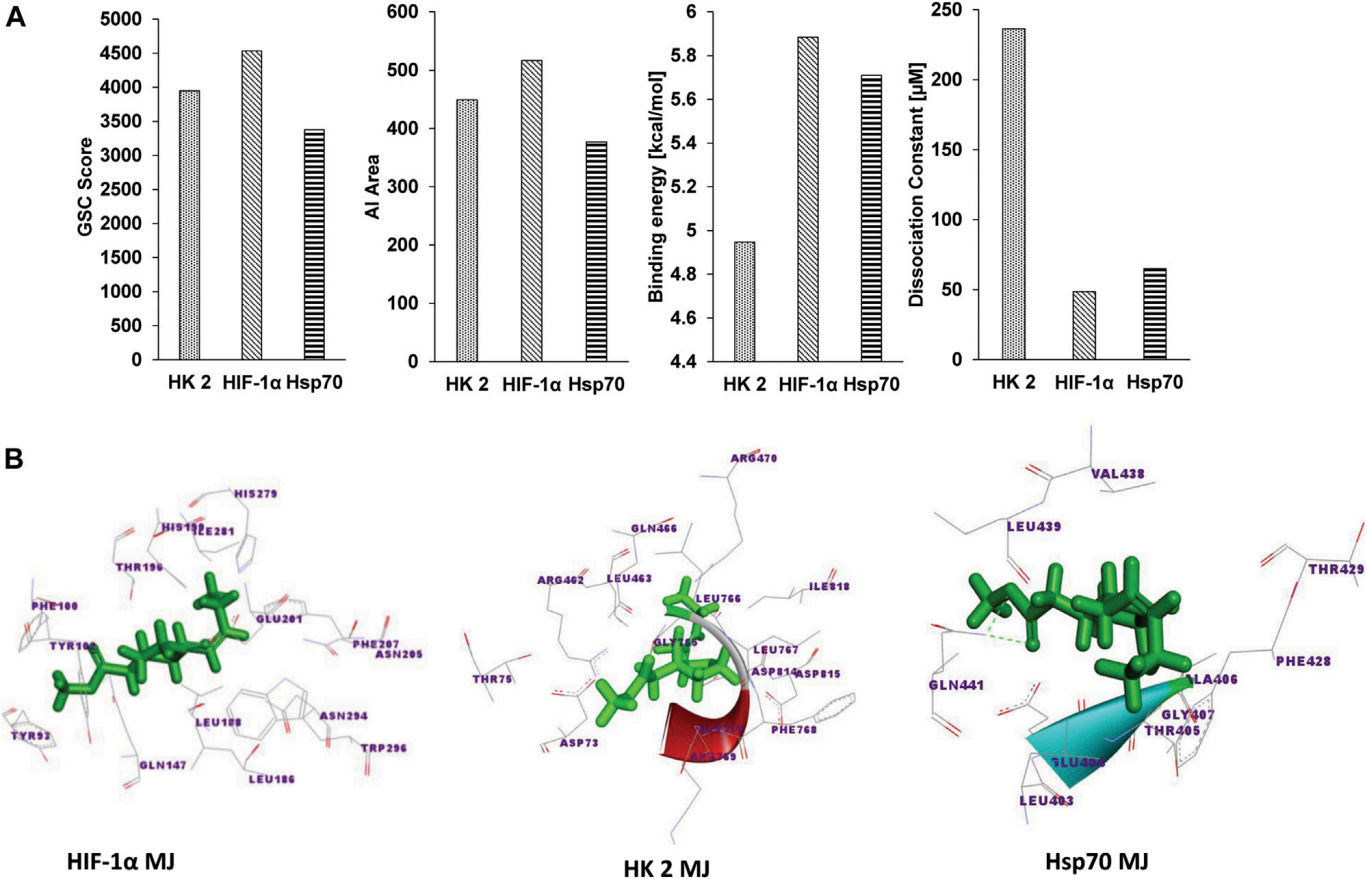
Docking analysis of MJ to Hsp70, HK 2, and HIF-1α. The docking strength of MJ to Hsp70, HK 2, and HIF-1α was analyzed on parameters of GSC score, AI area calculated by PatchDock, binding energy, and dissociation constant (Kd) calculated by YASARA **(A)**. The docked complexes were visualized by Discovery Studio 3.5, displaying the interaction of MJ to the indicated targets **(B)**.

Figure 1A shows the docking analysis results to characterize the molecular nature of the interaction of MJ to HIF-1α, HK 2, and Hsp70, based on the PatchDock server and YASARA, as described in the materials and methods. Parameters of the dissociation constant (Kd), GSC Score, binding energy, and AI area define the nature and strength of various interactions between target and ligand (Yadav et al., 2017b) were used for the docking analysis. Further, the Discovery studio 3.5 was used to visualize the docked complexes (Figure 1B). To characterize the molecular nature of the binding, we also deciphered the nature of interacting amino acids, H-bonds, active site amino acids, *vis-à-vis* identification of the prominent binding sites (Table 1). A detailed analysis of the parameters mentioned above revealed that in all targets (HIF-1α, HK 2 Hsp70), the interacting amino acids are located within the prominent binding sites metapocket based prediction. Most prominent binding site 1 displayed H-bonding between MJ and target protein HIF-1α and Hsp70. Additionally, other bonds identified to be involved in the interaction overwhelmingly included ionic and hydrophobic interactions and Van der Waals forces (data not shown). The primary interacting amino acids for all targets included (Table 1) the formation of H-bonds depending on the type and orientation of amino acids involved in the interactions (Yadav et al., 2017b). Interestingly, we observed variations in the interacting amino acids in each target’s case (Table 1), indicating that the formation of H-bonds was possibly independent of the commonness of contacting amino acids.

**TABLE 1:** Characterization of the docking of MJ to indicated targets.

Target protein	Interacted residues	Binding site no	MJ and protein atom involved in H-bonding	Interacting residues common with reported active binding sites
**HIF-1α**	TYR^102^ GLN^147^ LEU^186^ LEU^188^ THR^196^ HIS^199^ GLU^201^ PHE^207^ ARG^238^ GLN^239^ HIS^279^ ILE^281^ TRP^296^	1	Gln^147^:H, MJ:O2	ILE^281^ PHE^207^ THR^196^ TRP^296^
**HK 2**	ARG^69^ SER^70^ GLU^252^ GLY^253^ ARG^254^ LEU^463^ ALA^464^ HIS^467^ ARG^470^ ASP^814^ ILE^817^ ILE^818^	1	No H-bonding	Not available
**Hsp70**	LEU^403^ GLU^404^ THR^405^ ALA^406^ GLY^407^ GLY^408^ PHE^428^ THR^429^ THR^430^ TYR^431^ GLN^435^ VAL^438^ LEU^439^ ILE^440^ GLN^441^ ILE^474^ VAL^476^	1	Gln^441^:H; MJ:O3	Not available

### *In Vitro* Exposure to MJ Exerts an Inhibitory Action on Tumor Cell Metabolism, Expression of Metabolic Targets, and Survival

**FIGURE 2:**
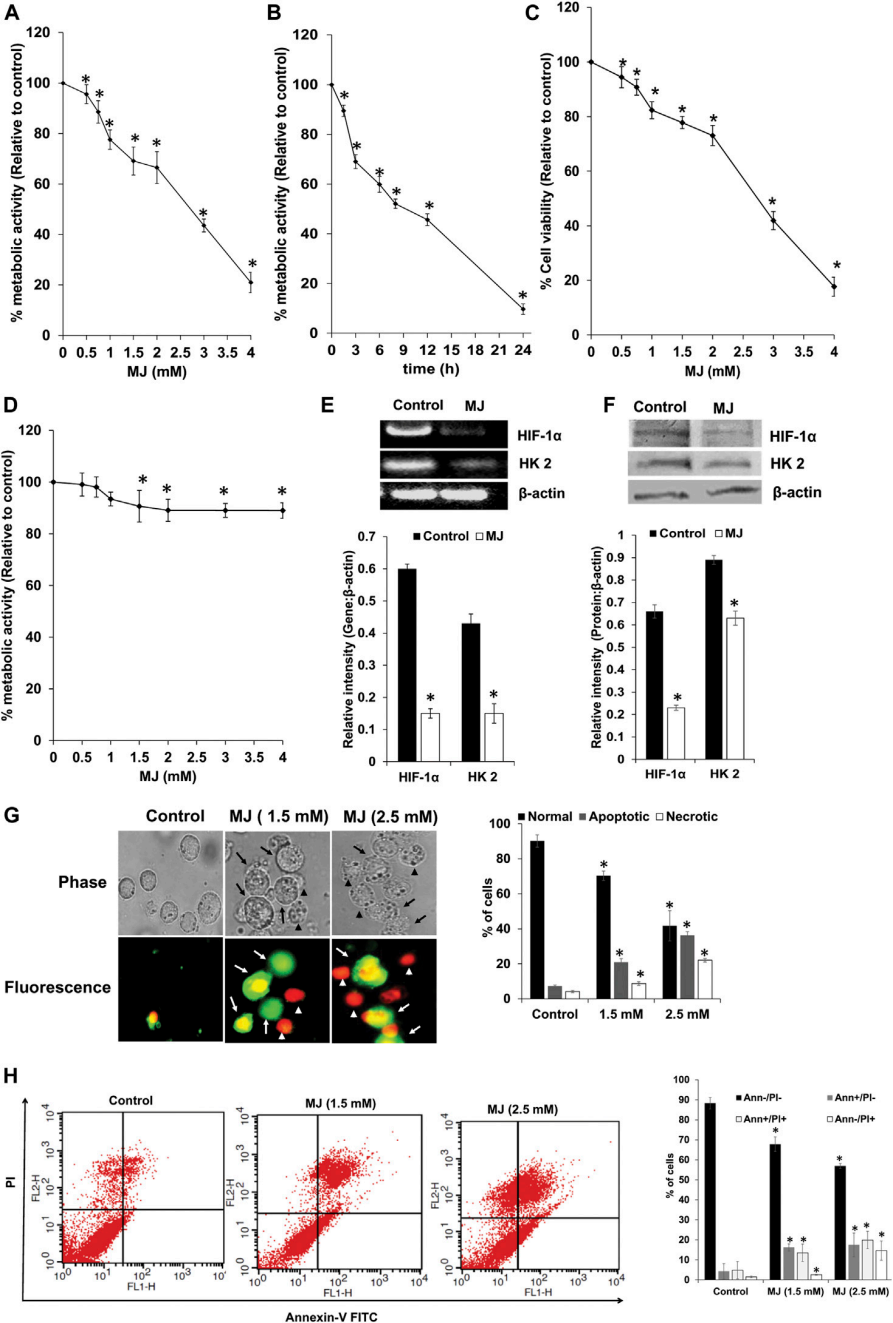
Effect of MJ on tumor cell metabolic activity, expression of metabolic molecules, tumor cell survival, apoptosis, and cytokine repertoire. Tumor cells (1 × 10^5^ cells/ml) were incubated in medium alone or containing the indicated concentrations of MJ **(A)** for 18 h or in medium with 2.5 mM MJ for the indicated time durations followed by estimation of metabolic activity **(A,B)**, and cell survival **(C)** as described in the materials and methods. Thymocytes (1 × 10^5^ cells/ml) were incubated in medium with or without the indicated concentration of MJ for 18 h, followed by estimation of metabolic activity **(D)**. Cell lysates of control and MJ-treated (2.5 mM) tumor cells (1 × 10^5^ cells/ml) were examined for gene and protein expression of the indicated metabolic molecules by RT-PCR and western blotting, respectively. Bands shown are from a representative experiment out of three independent experiments with a similar pattern. The accompanying bar diagrams are the densitometry analysis of the bands **(E,F)**. Tumor cells (1 × 10^5^ cells/ml) were incubated in medium alone or containing the indicated concentration of MJ for 18 h followed by estimation of cell death by fluorescence microscopy **(G)** and flow cytometry **(H)**, as described in the materials and methods. Arrows and arrowheads indicate apoptotic and necrotic cells, respectively in the microscopic images. Flow cytometric and microscopic fluorescence images are from a representative experiment out of three independent experiments with a similar pattern. Values shown are bar diagrams are mean ± SD of three independent experiments. *p < 0.05 vs. respective control.

HIF-1α, HK 2, and Hsp70 play an indispensable role in regulating survival and metabolism. Hence, next, we estimated survival and metabolic activity in MJ-treated tumor cells. Therefore, the dose and time kinetics of tumor cells’ response on their metabolic activity, following *in vitro* exposure of MJ was examined. As shown in Figures 2A,B, MJ treatment of tumor cells (1 × 10^5^ cells/ml) resulted in the significant inhibition of the metabolic activity in a dose and time-dependent manner compared to the untreated control. IC_50_ values were calculated from both MTT (IC_50_: 2.4 mM) and cell proliferation assays (IC_50_: 2.1 mM). Hence, in all subsequent experiments, tumor cells were incubated in medium alone (control) or containing MJ (2.5 mM) for 18 h, except for estimation of ROS expression, where the tumor cells were exposed to the same concentration of MJ for 2 h and 4 h for the evaluation of mitochondrial membrane potential. Trypan blue dye exclusion test was carried out to analyze the anti-survival effect of MJ on tumor cells *in vitro*. Tumor cells (1 × 10^5^ cells/ml) incubated in medium alone or containing the indicated concentrations of MJ showed a significant dose-dependent decline in tumor cell survival compared to the untreated control (Figure 2C). The tumor cell-specific cytotoxic action of MJ was determined by incubating thymocytes (1 × 10^5^ cells/ml) in a medium with or without the indicated concentrations of MJ for 18 h followed by estimation of the metabolic activity (Figure 2D). Exposure of thymocytes to various MJ concentrations had little effect on their metabolic activity, indicating tumor-cell-specific antimetabolic action of MJ.

As we observed metabolic inhibitory action of MJ, which could be due to inhibition of HIF-1α and HK 2 by MJ; hence next, we examined the expression pattern of HIF-1α and HK 2 at the transcriptional and translational level in control and MJ-treated cells. The results are shown in Figures 2E,F. Treatment of the tumor cells with MJ resulted in a significant decline in the expression of HIF-1α and HK 2 compared to control.

To understand the mode of cell death, control and MJ-treated tumor cells were stained with Annexin V/PI for determining the mode of induction of cell death by fluorescence microscopy (Figure 2G) and flow cytometric analysis (Figure 2H). Treatment of tumor cells with MJ resulted in a significant increase in the population of cells displaying features of apoptotic and necrotic modes of cell death compared to the untreated control.

### MJ Alters Mitochondrial Functions, ROS Production, and Expression of Cell Survival Regulatory Molecules, and Production of Cytokines

**FIGURE 3:**
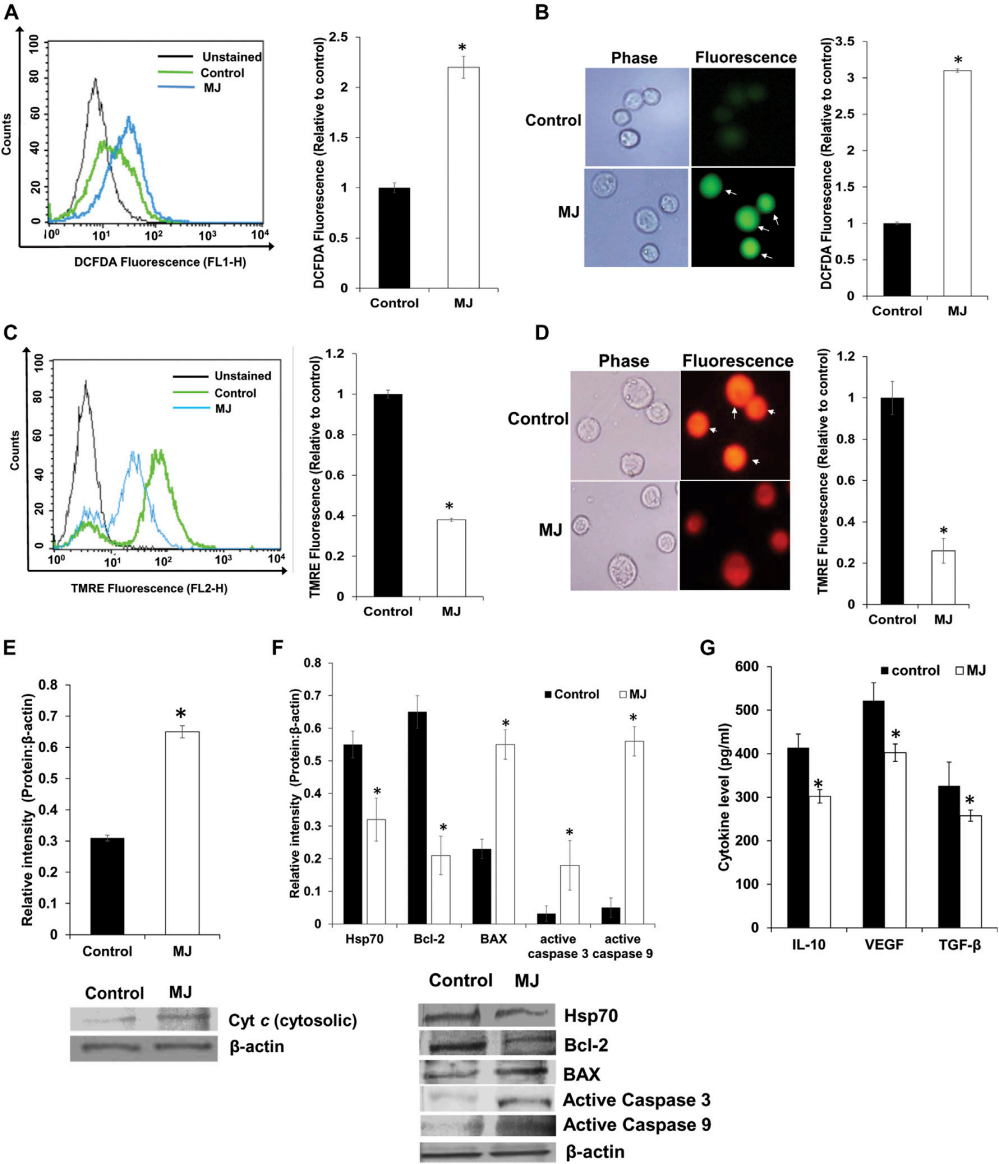
Effect of MJ on the expression of ROS, mitochondrial membrane potential and expression of cytosolic Cyt *c* and cell survival regulatory molecules. Tumor cells (1 × 10^5^ cells/ml) were incubated in medium alone or containing MJ (2.5 mM) for 2 h, followed by estimation of ROS expression by flow cytometry **(A)** and fluorescence microscopy **(B)**. The mitochondrial membrane potential was estimated following 4 h of MJ (2.5 mM) treatment by flow cytometry **(C)** and fluorescence microscopy **(D)**. The cytosolic fraction of the control and MJ (2.5 mM) treated tumor cells (1 × 10^5^ cells/ml) following 18 h of incubation in the respective treatments were analyzed for the Cyt *c* by Western blotting **(E)**. The flow cytometric **(A,C)**, fluorescence microscopic **(B,D)**, and Western blotting **(E)** images are from a representative experiment out of three independent experiments with a similar pattern. Arrows indicate cells positive for DCFDA **(B)** and TMRE staining **(D)**, respectively. Accompanying bar diagrams **(A–E)** are mean ± SD of quantitative values of the flow cytometric analysis and densitometry of fluorescence, and Western blots, respectively. *p < 0.05 vs. respective control. Tumor cells (1 × 10^5^ cells/ml) of control and MJ-treated (2.5 mM) groups were also analyzed for the expression of the indicated cell survival regulatory proteins by Western blotting as described in the materials and methods **(F)**. Bands **(F)** shown are from a representative experiment out of three independent experiments with a similar pattern. The accompanying bar diagrams are the densitometry analysis of the bands **(F)**. Tumor cells (1 × 10^5^ cell/ml) were incubated in medium alone or containing MJ (2.5 mM) for 18 h followed by estimation of the indicated cytokines in the culture supernatant by ELISA **(G)** as described in the materials and methods. Values shown are bar diagrams are mean ± SD of three independent experiments. *p < 0.05 vs. respective control. Values shown are mean ± SD *p < 0.05 vs. respective control.

Here, we checked the mitochondrial membrane potential and ROS production to evaluate the role of MJ-mediated mitochondrial dysfunction in apoptosis induction. Control and MJ (2.5 mM) treated tumor cells were examined for expression of ROS by flow cytometry (Figure 3A) and fluorescence microscopy (Figure 3B) as described in the materials and methods. Treatment of tumor cells with MJ resulted in a significant increase in ROS expression compared to the untreated control. Because MJ can cause dissociation of HK 2 from the mitochondrial membrane, which leads to depolarization of mitochondria potential (Goldin et al., 2008), we also checked if MJ could cause mitochondrial membrane depolarization of DL cells. Tumor cells (1 × 10^5^ cells/ml) were incubated in medium alone or containing MJ (2.5 mM) for 4 h followed by staining with TMRE and analysis of cells by flow cytometry (Figure 3C) and fluorescence microscopy (Figure 3D) for analyzing the effect of MJ treatment on mitochondrial membrane potential as described in the materials and methods. Treatment of tumor cells with MJ resulted in a significant decline of mitochondrial potential as indicated by the decrease of TMRE fluorescence compared to untreated control. Since a reduction of TMRE fluorescence is an indication of a declined mitochondrial membrane potential, which is accompanied by the release of Cyt *c* (Esteras et al., 2020), we also checked the level of cytosolic Cyt *c* in control and MJ-treated tumor cells. The results are shown in Figure 3E. Treatment of tumor cells with MJ resulted in a significantly enhanced level of cytosolic Cyt *c* as compared to the untreated control.

As MJ treatment caused an elevated level of cytosolic Cyt c, next, we checked if it was associated with modulation in the expression of apoptosis regulatory molecules. Treatment of tumor cells with MJ (2.5 mM) for 18 h resulted in a significant decline in the expression of Bcl-2 compared to control. In contrast, the expression of active caspases 3 and 9, and BAX was significantly enhanced compared to the respective control (Figure 3F). Further, we also examined the level of Hsp70 in control and treated tumor cells due to its critical role in regulating apoptotic and necrotic cell death (Boudesco et al., 2018). Interestingly, we noticed a significant decline in the expression of Hsp70 in MJ-treated tumor cells compared to untreated control (Figure 3F).

As cytokines play an essential role in the regulation of cell survival and metabolic signaling, next, we checked if MJ treatment of tumor cells could modulate cytokine expression. Tumor cells (1 × 10^5^ cells/ml) were incubated in a medium with or without MJ (2.5 mM) for 18 h followed by estimation of IL-10, VEGF, and TGF-β in the culture supernatant by ELISA as described in the materials and methods. The results are shown in Figure 3G, treatment of tumor cells with MJ resulted in a significant decline in the level of IL-10, VEGF, and TGF-β compared to the respective control.

### Alteration of pH Homeostasis in Methyl Jasmonate Treated Tumor Cells

**FIGURE 4:**
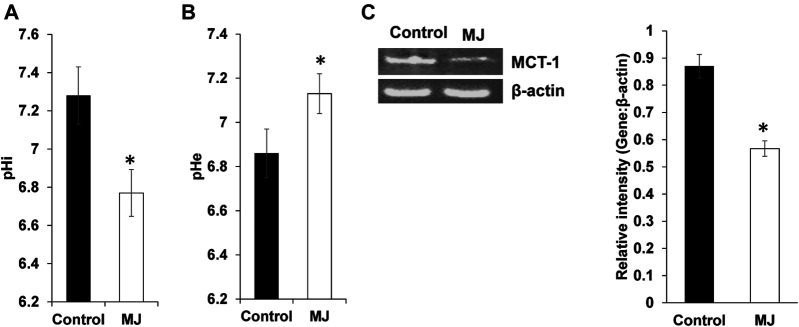
MJ modulates pH homeostasis of tumor cells. Tumor cells (1 × 10^5^ cell/ml) were incubated in medium alone or containing MJ (2.5 mM) for 18 h followed by estimation of pHi **(A)** and pHe **(B)** as described in the materials and methods. Values shown in the bar diagrams **(A,B)** are mean ± SD of three independent experiments. *p < 0.05 vs. respective control. Expression of pH regulator MCT-1 was analyzed in the cell lysate of control and MJ-treated tumor cells (1 × 10^5^ cell/ml). Bands **(C)** shown are from representative experiments out of three with a similar pattern. **(C)**. The accompanying bar diagrams are densitometric analysis of the bands **(C)**. Values shown are mean ± SD. *p < 0.05 vs. respective control.

Culture supernatant of control and MJ treated tumor cells were assayed for pHe (extracellular pH), whereas their cell lysates for pHi as described in materials and methods. Results are shown in Figures 4A,B, treatment of tumor cells with MJ resulted in a significant decline in pHi along with an increase of pHe compared to respective untreated control. To understand the underlying mechanism, the expression of MCT-1, a pH regulator responsible for lactate transport, was estimated. As shown in Figure 4C, MJ treated tumor cells displayed inhibited expression of MCT-1 compared to untreated control.

### Methyl Jasmonate Alters the Chemosensitivity of Tumor Cells

**FIGURE 5:**
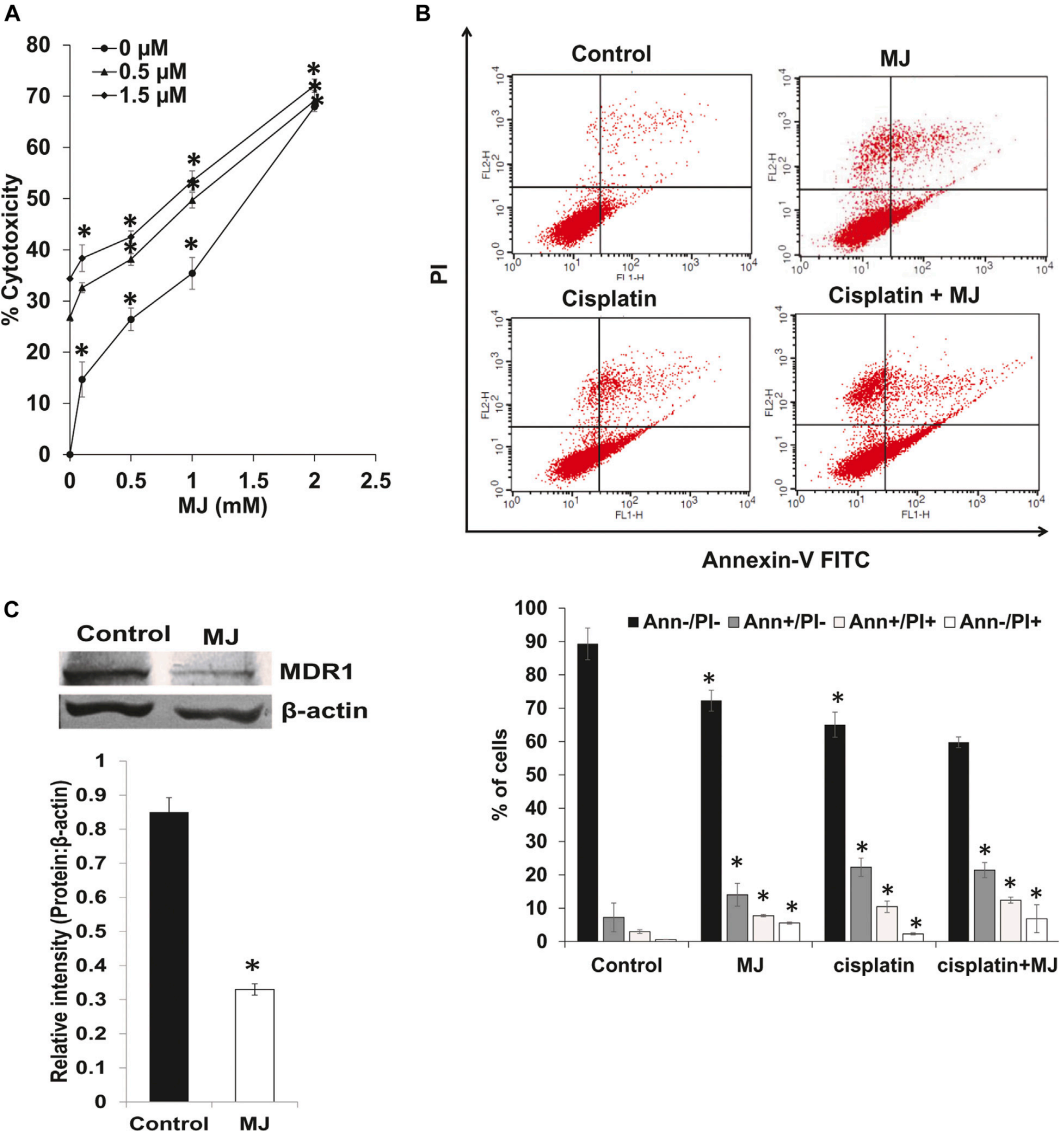
MJ enhances chemosensitivity of tumor cells. Tumor cells (1 × 10^5^ cell/ml) cells were incubated in medium alone or containing MJ in the presence or absence of cisplatin for 18 h followed by estimation of the cytotoxicity **(A)** as described in the materials and methods. Tumor cells (1 × 10^5^ cell/ml) incubated in medium alone or containing MJ in the presence or absence of cisplatin for 18 h were analyzed for apoptotic cell population by flow cytometry using Annexin-V/PI staining as described in the materials and methods. The flow cytometric image **(B)** is from a representative experiment out of three independent experiments with similar results. The bar diagram accompanying the flow cytometric images depicts the mean ± SD of the % of cells. *p < 0.05 vs. respective control. In a parallel set of experiments, the cell lysates of control and MJ-treated tumor cells (1 × 10^5^ cell/ml) were analyzed for expression of MDR1. Bands **(C)** are from a representative experiment out of three with a similar pattern. The accompanying bar diagrams are densitometric analysis of the bands **(C)**. Values shown are mean ± SD. *p < 0.05 vs. respective control.

We also analyzed the effect of MJ on the cytotoxicity of cisplatin and the expression of MDR1. Tumor cells (1 × 10^5^ cells/ml) were incubated in medium alone or containing MJ with or without the indicated concentrations of cisplatin for 18 h followed by estimation of cytotoxicity (Figure 5A). To evaluating the effect of MJ on the tumor cell killing ability of cisplatin, tumor cells (1 × 10^5^ cells/ml) were incubated in medium alone or containing MJ in the presence or absence of cisplatin and flow cytometric examination of cell death (Figure 5B) was carried out. Incubation of tumor cells in the presence of MJ and cisplatin significantly augmented tumor cell death depending upon the concentrations of MJ compared to control and tumor cells incubated in a medium containing only cisplatin or MJ. A similar pattern was observed concerning the induction of cell death as analyzed by flow cytometry. Tumor cells incubated in a medium containing both cisplatin and MJ had a significantly higher population of apoptotic and necrotic cells than control and cells incubated either with medium or containing cisplatin MJ (Figure 5B). To understand the mechanism of the increased chemosensitivity of MJ-treated tumor cells, we also examined the expression of MDR1. The results are shown in Figure 5C. MDR1 expression was significantly declined in MJ-treated tumor cells compared to the untreated control.

## Discussion

The present investigation observations indicate that MJ exerts potent cytotoxic action on tumor cells of T-cell origin. Further, the antimetabolic action of MJ was accompanied by an inhibited expression of critical metabolic targets in the tumor cells, including HIF-1α, and HK 2. Interestingly, Li et al., (2017) have shown the inhibitory effect of MJ on the expression of HK 2 at mRNA and protein levels, indicating the possible role of impaired transcriptional and translational regulation (Li et al., 2017). Similarly, we also observed decreased expression of HK 2 at both transcriptional and translational levels in our study. The same could possibly due to MJ-mediated suppression of HIF-1α expression. The MJ-dependent inhibited expression of HIF-1α might also be responsible for the down-regulated expression of Hsp70 (Huang et al., 2009; Tsuchida et al., 2014). More importantly, our study suggests the possible involvement of MJ-dependent decreased TGF-β level in the inhibition of HIF-1α protein as TGF-β has been reported in the induction of HIF-1α level via enhancing its stabilization through declined prolyl hydroxylase 2 (PHD2) level (Han et al., 2013). Moreover, several cancer-promoting factors, such as Bclaf1, PRMT1, and angiotensin II, have been reported in the transcriptional regulation of HIF-1α (Lafleur et al., 2014). Hence, the role of these molecules cannot be ruled out in the MJ-mediated inhibition of HIF-1α expression at the transcriptional level, which necessitates further investigation. Besides, our study also ushers a future direction to evaluate the role of MJ-modulated proteasomal degradation in the inhibited expression of its target proteins.

Indeed, all of these molecules play a decisive role in regulating the tumor cell metabolism (Goldin et al., 2008; Martinez-Outschoorn et al., 2017; Boudesco et al., 2018; Pezzuto and Carico, 2018). Among these metabolic targets, HIF-1α is an upstream master regulator of tumor metabolism (Pezzuto and Carico, 2018). Further, hypoxia-induced modulation of HIF-1α is shown to be associated with the detachment of HK 2 from the VADC (Cai et al., 2019). Thus, MJ-triggered inhibition of HIF-1α could be one of the likely causes of inhibited downstream metabolic mediators' expression. Likewise, HIF-1α also regulates hypoxia and rearranged metabolism associated expression of cell survival regulatory Bcl-2, BAX, and caspases (Pezzuto and Carico, 2018). Moreover, we also observed inhibition in the expression of Hsp70 in MJ-treated tumor cells, which again indicates that MJ leads to impaired tumor cell survival via modulation of multiple stakeholders. Indeed, Hsp70 is reported to be indispensable for tumor cell metabolism, survival, and proliferation (Boudesco et al., 2018). Studies report the interaction of HIF-1α and Hsp70 in modulating mitochondrial functions, tumor cell metabolism, and survival (Julia et al., 2017; Boudesco et al., 2018; Li et al., 2019; Nagao et al., 2019).

Docking analysis of this investigation also indicated that MJ displayed a strong binding to HIF-1α. Indeed, reports suggest that binding of metabolic inhibitors to HIF-1α can render its inactivation (Pezzuto and Carico, 2018). Furthermore, the docking analysis results also demonstrated that MJ could bind to HK 2 at the prominent binding site, indicating the additional capability of MJ to render HK 2 inactive of its catalytic activity. Inhibition of HIF-1α has been reported to cause an inhibition of HK 2 and hence manifest an intense antimetabolic scenario (Nagao et al., 2019). The consequent depletion of ATP availability further hampers HK activity, as in the absence of an optimum concentration of ATP, the catalyzing speed of HK 2 gets compromised (Mathupala et al., 2006).

Nevertheless, it is also reported that under such conditions, HK 2 is more susceptible to glucose 6-phosphate, the product-dependent inhibition (Mathupala et al., 2006). Ours is the first report of its kind, providing *in silico* based evidence to demonstrate direct physical interaction of MJ with HIF-1α and HK 2, which suggests novel mechanisms of the antimetabolic action of MJ. Nevertheless, as MJ binds to meta-pocket site 1 of these two critical metabolic targets, the same strongly indicates MJ to be stoichiometrically a preferred drug for inhibition of HIF-1α and HK 2. This part of the study’s observations suggests the potential to explore MJ as an inhibitor of HIF-1α. It needs to be precisely explored how MJ alters HIF-1α expression at mRNA expression, protein synthesis, protein degradation and dimerization, DNA binding, and transcriptional activity levels.

We also compared the docking of MJ to its primary target HK 2 *vis a vis* to that of another HK2 inhibitor, lonidamide. Lonidamine and MJ bind to the same binding pocket that is 1 as seen by Metapocket 2.0 (data not shown). Hence, we may conclude that both drugs bind to the same active pocket of HK 2, on the contrary glucose, and glucose-6-PO_4_ bind to binding pocket 2; therefore, we may infer that since the binding takes place in different active pockets, there is no competition between glucose and MJ. In a previous docking analysis study of 3-BP, which also inhibits HK 2 (Yadav et al., 2017b), we had reported that 3-BP binds to HK 2, leading to conformational changes of the active sites (Yadav et al., 2017b). However, the present study reveals that the GSC score and Kd of MJ indicate a more durable binding than 3-BP. This difference could also be attributed to the difference in the binding pockets involved and the interacting amino acids. The more durable binding of MJ to HK 2 is also apparent in terms of the inhibitory kinetics of MJ on metabolism, which reached its optimal level within 6–10 h in the present study compared to 24–48 h for 3-BP as reported previously (Gupta et al., 2020).

We also observed MJ-dependent downregulated expression of Hsp70, which plays a crucial role in supporting tumor cell survival in multiple ways (Lazarev et al., 2018). Hence the same, in turn, could be responsible for the observed inhibition of tumor cell survival. The expression of Hsp70, in turn, is under the regulation of HIF-1α (Pezzuto and Carico, 2018). Further, our docking analysis study also showed a strong binding ability of MJ to the prominent binding site of Hsp70. This indicates the likelihood of the ability of MJ not only to inhibit the expression of downstream metabolic targets HIF-1α but also by its ability to interact with their prominent binding site physically, thereby compromising their activity.

Interestingly, HIF-1α, HK 2, and Hsp70 share similar amino acid sequences LEU, GLU, and ILE, which allow MJ binding at the most prominent active pocket. We also identified and characterized COX2, GAPDH, and PDH as potential targets of MJ based on a similar method. They also share same common residues at the prominent active binding site (Supplementary Table S4). Therefore, our *in silico* findings also suggests a need to perform MS-proteomic and CESTA analyses to identify and characterize novel targets of MJ.

Notably, our results showed cytotoxic action of MJ against tumor cells at relatively higher concentrations. Similarly, studies conducted on various other neoplastic cells also show that the cytotoxic activity of MJ is apparent only in the mM concentration ranges (Cesari et al., 2014; Zhang et al., 2015; Yousefi et al., 2020). There could be multiple reasons underlying this observation. Considering the volatile nature of MJ, it is likely that the same could be one of the causes for its higher effective concentration. Hence, the actual biological response modifying concentration may be much lower than the concentration used for the treatment. However, the docking analysis indicated a strong binding of MJ to its targets. Hence, the possibility of its weak binding to targets or non-selectivity is less likely. Additionally, other studies have reported strong binding of MJ to HK 2, its primary target, leading to inhibition of tumor metabolism (Li et al., 2017). Further, the treatment concentration can be decreased by enhancing the stability of MJ through chemical modifications or synthesis of MJ-derived non-volatile derivatives, which could constitute promising anticancer regimens due to improved stability and ability to bind to multiple target molecules.

Further, a survey of the literature indicates that this is the first report demonstrating the inhibitory action of MJ on the expression of MCT-1 in neoplastic cells. The augmented glycolysis of neoplastic cells generates a high amount of lactate in tumor cells, which can cause cytosolic acidification, capable of inhibiting cellular functions and inducing cell death (Yadav et al., 2017a). Therefore, to prevent cytosolic acidification, tumor cells display a highly up-regulated expression of pH regulators like MCT-1, which transports lactate across the cell membrane to the external milieu (Yadav et al., 2017a). Thus, an inhibited expression of MCT-1 in MJ-treated DL cells could have interfered with their pH homeostasis, reversing MCT-1 dependent relative alkalization of cytosol. Interestingly, HIF-1α is also reported as an essential regulator of MCT-1 in neoplastic cells (Miranda-Gonçalves et al., 2016). In concurrence with the antimetabolic effects, treatment of tumor cells with MJ was also observed to manifest anti-survival and pro-apoptotic action. Inhibition of tumor cell survival could depend on declined energy production and augmented expression of ROS in MJ treated tumor cells. Decreased ATP levels and increased ROS concentration have been reported to be a key player mediating the cytotoxic action of MJ against other types of neoplastic cells (Zhang et al., 2016; Yousefi et al., 2020).

Nevertheless, accompanied by the increased expression of ROS, we also observed a declined mitochondrial membrane potential following MJ treatment of tumor cells, indicating mitochondrial implication in the induction of cell death. This notion was also corroborated by the observation showing augmented cytosolic Cyt *c*, indicating its enhanced release following altered mitochondrial membrane permeability. We also observed augmented expression of apoptosis-regulating molecules like active caspase 3 and 9 and BAX along with downregulation of antiapoptotic Bcl-2, implicating the role of these molecules in MJ-dependent induction of cell death. Our observations are in tune with other reports indicating the role of mitochondrial-dependent cell death following MJ treatment of tumor cells of other origins (Cesari et al., 2014; Li et al., 2017).

Moreover, we observed an altered repertoire of tumor growth regulatory cytokines in MJ treated tumor cells. The declined expression of tumor growth-promoting IL-10, TGF-β, and VEGF could depend on the altered expression of their upstream regulators: HIF-1α and Hsp70 (Yadav et al., 2017a). The cytokines mentioned above are also reported to regulate molecules involved in apoptosis cascade and metabolic processes. Another study has indicated synergy between IL-10 and TGF-β in modulating mitochondrial membrane potential and ROS generation via alteration of metabolic programming (Soukupova et al., 2017). Nevertheless, signaling via cytokines like TGF-β can alter mitochondrial bioenergetic homeostasis, including modulation of membrane potential (Yoon et al., 2005; Casalena et al., 2012). Interestingly, a recent study has shown the MCT-1 regulatory action of VEGF in acute myeloid leukemia cells (Lopes-Coelho et al., 2017); hence the down-regulated expression of VEGF could also be a significant factor for the declined expression of MCT-1. To best of our knowledge, this is the first report regarding the modulatory action of MJ on the production of IL-10, and TGF-β by tumor cells.

Additionally, we also observed the chemosensitizing ability of MJ against T lymphoma cells. The increased chemosensitization of tumor cells to cisplatin was accompanied by the downregulated expression of MDR1. Further, the expression of MDR1 has been reported to be regulated by cytokines like IL-10 and TGF-β (Mukherjee et al., 2013), and HIF-1α (Comerford et al., 2002). Moreover, HIF-1α and Hsp70 have been reported to regulate the expression and activity of MDR1 (Yadav et al., 2017a). Indeed, overexpression of MDR1 in neoplastic cells is linked to accelerated metabolism (Zhao et al., 2013). Moreover, a study by Cen et al., has also indicated ROS's implication in the modulated expression of MDR regulating proteins (Cen et al., 2016).

## Conclusion

**FIGURE 6:**
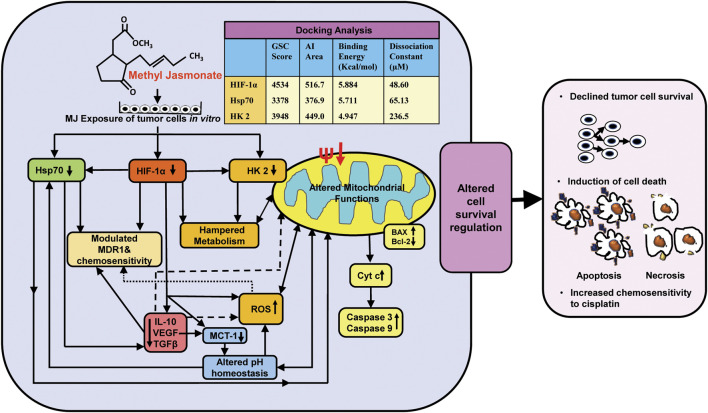
Summary of the molecular mechanisms underlying cytotoxic, antimetabolic, and chemosensitizing action of MJ against T cell lymphoma. The figure presents a summary of the molecular events implicated in the cytotoxic, antimetabolic, and chemosensitizing action of MJ against DL cells *in vitro* and docking analysis of MJ with indicated metabolic and cell survival regulatory targets.

The findings of the present investigation shed new light regarding molecular mechanisms of the cytotoxic action of MJ against T cell lymphoma in conjunction with inhibited metabolism, altered pH homeostasis, increased ROS generation, hampered mitochondrial functions, and modulated expression of cell survival, metabolism, chemoresistance regulating molecules along with direct docking ability of MJ to the prominent binding sites of critical metabolism and cell survival regulatory molecules HIF-1α, HK 2, and Hsp70 (Figure 6). Overall, the present investigation will significantly contribute to designing anticancer therapeutic regimens using MJ against malignancies of T-cell origin.

## Data Availability

The raw data supporting the conclusions of this article will be made available by the authors, without undue reservation.
