# Supplementary material for: Methyl Jasmonate Cytotoxicity and Chemosensitization of T Cell Lymphoma In Vitro Is Facilitated by HK 2, HIF-1α, and Hsp70: Implication of Altered Regulation of Cell Survival, pH Homeostasis, Mitochondrial Functions
Source: Front Pharmacol. 2021 Feb 26;12:628329. doi: 10.3389/fphar.2021.628329 (PMC7954117; doi:10.3389/fphar.2021.628329)
Supplement: Supplementary file 1 [file table1.docx]

**Supplementary Table. 1 Primer sequences for RT-PCR analysis**

| **Genes** | **Primer sequences** |
| --- | --- |
| **HIF-1α** | F-5’-CTCAAAGTCGGACAGCCTCA-3’; R-5’-CCCTGCAGTAGGTTTCTGCT-3' |
| **HK 2** | F-5’-TGATCGCCTGCTTATTCACGG-3’; R-5’-AACCGCCTAGAAATCTCCAGA-3' |
| **MCT1** | F-5’-CCATTGTGGAATGCTGTCCT-3’; R-5’-CCTACTTCTTTCCCCCATCC-3' |
| **β-Actin** | F-5’GGCACAGTGTGGGTGAC-3’; R-5’-CTGGCACCACACCTTCTAC-3' |
