# Supplementary material for: Methyl Jasmonate Cytotoxicity and Chemosensitization of T Cell Lymphoma In Vitro Is Facilitated by HK 2, HIF-1α, and Hsp70: Implication of Altered Regulation of Cell Survival, pH Homeostasis, Mitochondrial Functions
Source: Front Pharmacol. 2021 Feb 26;12:628329. doi: 10.3389/fphar.2021.628329 (PMC7954117; doi:10.3389/fphar.2021.628329)
Supplement: Supplementary file 2 [file table2.docx]

**Supplementary Table. 2 Details of the retrieved target protein molecules**

| **Target proteins** | **PDB advance BLAST details** |
| --- | --- |
| **HIF-1α** | 5JWP, DOI: 10.2210/pdb5JWP/pdb |
| **HK 2** | 5HEX. DOI: 10.2210/pdb5HEX/pdb |
| **Hsp70** | 6JPV, DOI: 10.2210/pdb6JPV/pdb |
