# Supplementary material for: Methyl Jasmonate Cytotoxicity and Chemosensitization of T Cell Lymphoma In Vitro Is Facilitated by HK 2, HIF-1α, and Hsp70: Implication of Altered Regulation of Cell Survival, pH Homeostasis, Mitochondrial Functions
Source: Front Pharmacol. 2021 Feb 26;12:628329. doi: 10.3389/fphar.2021.628329 (PMC7954117; doi:10.3389/fphar.2021.628329)
Supplement: Supplementary file 3 [file table3.docx]

**Supplementary Table. 3 PubChem ID**

| **Name of Ligand** | **PubChem CID** |
| --- | --- |
| **MJ** | 5281929 |
