# Supplementary material for: Methyl Jasmonate Cytotoxicity and Chemosensitization of T Cell Lymphoma In Vitro Is Facilitated by HK 2, HIF-1α, and Hsp70: Implication of Altered Regulation of Cell Survival, pH Homeostasis, Mitochondrial Functions
Source: Front Pharmacol. 2021 Feb 26;12:628329. doi: 10.3389/fphar.2021.628329 (PMC7954117; doi:10.3389/fphar.2021.628329)
Supplement: Supplementary file 4 [file table4.docx]

**Supplementary Table. 4**

| **Target Protein (PDB ID)** | **Interacted residues** |
| --- | --- |
| **GAPDH (1u8f)** | ASN^9^ GLY^10^ PHE^11^ GLY^12^ ASN^34^ ASP^35^ PRO^36^ PHE^37^ GLU^79^ ARG^80^ PRO^82^ ILE^85^ SER^98^ THR^99^ VAL^101^ PHE^102^ |
| **COX2 (5f19)** | CYS^36^ HIS^39^ PRO^40^ CYS^41^ GLN^42^ ARG^44^ GLY^45^ VAL^46^ CYS^47^ TYR^130^ LYS^137^ LEU^152^ PRO^153^ GLN^461^ GLU^465^ LYS^468^ ARG^469^ |
| **PDH (3exe)** | ILE^57^ ILE^58^ ARG^59^ GLY^60^ PHE^61^ GLU^108^ LEU^109^ THR^110^ GLY^111^ GLY^119^ LYS^120^ GLY^121^ GLY^122^ SER^123^ MET^124^ HIS^125^ |
